# A cautionary note regarding count models of alcohol consumption in randomized controlled trials

**DOI:** 10.1186/1471-2288-7-9

**Published:** 2007-02-15

**Authors:** Nicholas J Horton, Eugenia Kim, Richard Saitz

**Affiliations:** 1 Department of Mathematics and Statistics, Smith College, Northampton, MA, USA; 2 Clinical Addiction Research and Education (CARE) Unit, Section of General Internal Medicine, Boston Medical Center and Boston University School of Medicine, Boston, MA, USA; 3 Youth Alcohol Prevention Center, Boston University School of Public Health, Boston, MA, USA; 4 Department of Epidemiology, Boston University School of Public Health, Boston, MA, USA

## Abstract

**Background:**

Alcohol consumption is commonly used as a primary outcome in randomized alcohol treatment studies. The distribution of alcohol consumption is highly skewed, particularly in subjects with alcohol dependence.

**Methods:**

In this paper, we will consider the use of count models for outcomes in a randomized clinical trial setting. These include the Poisson, over-dispersed Poisson, negative binomial, zero-inflated Poisson and zero-inflated negative binomial. We compare the Type-I error rate of these methods in a series of simulation studies of a randomized clinical trial, and apply the methods to the ASAP (Addressing the Spectrum of Alcohol Problems) trial.

**Results:**

Standard Poisson models provide a poor fit for alcohol consumption data from our motivating example, and did not preserve Type-I error rates for the randomized group comparison when the true distribution was over-dispersed Poisson. For the ASAP trial, where the distribution of alcohol consumption featured extensive over-dispersion, there was little indication of significant randomization group differences, except when the standard Poisson model was fit.

**Conclusion:**

As with any analysis, it is important to choose appropriate statistical models. In simulation studies and in the motivating example, the standard Poisson was not robust when fit to over-dispersed count data, and did not maintain the appropriate Type-I error rate. To appropriately model alcohol consumption, more flexible count models should be routinely employed.

## Background

Count outcomes are common in randomized studies of alcohol treatment. Subjects may be queried about their daily consumption of alcohol, measured as a number of drinks over a recent period [1] (typically 30 days), and these values are used to estimate average drinking per day. In this setting, estimating differences between treatment group and control group is of primary interest.

A challenge in modeling consumption outcomes is to appropriately account for the distribution of drinking. These distributions are characterized by a large number of zeros (abstinent subjects) along with a long right tail (heavy drinking subjects). An extensive literature describes models for counts [2-8], and they have been commonly applied in economic analyses, traffic accidents, and health services utilization. Many routines are now available in general purpose statistical software (e.g. Stata) [8]. A natural model for counts is the single-parameter Poisson distribution. One disadvantage of the Poisson is that it makes strong assumptions regarding the distribution of the underlying data (in particular, that the mean equals the variance). While these assumptions are tenable in some settings, they are less appropriate for alcohol consumption. Extensions of the Poisson, such as the over-dispersed Poisson, negative binomial and two stage (hurdle) or zero inflated models have been proposed [2-5].

Our methods are motivated by the analysis of the ASAP (Addressing the Spectrum of Alcohol Problems) study, a randomized clinical trial comparing a brief motivational interview to usual care for a sample of inpatients with unhealthy alcohol use at an urban hospital [9]. These subjects were followed to see if there were differences in drinking outcomes that could be attributed to randomized group assignment.

In this paper, we will demonstrate the limitations of the standard Poisson model in the presence of over-dispersion. We begin by describing several count models for alcohol outcomes, compare their performance in a series of simulated randomized trials, apply them to the ASAP study, and conclude with some general recommendations.

## Methods

### Statistical methods for the analysis of count outcomes

We begin by introducing notation to be used throughout. Let *Y*_*ij *_denote the number of events for the *j*th subject (*j *= 1,..., *n*_*i*_) in the *i*th group (*i *= 1, 2), where *n*_*i *_is the number of subjects in the *i*th group. Typically in a randomized trial *n*_1 _and *n*_2 _are approximately equal.

The Poisson distribution is one of the simplest models for count data. Let *λ*_*ij *_indicate the average number of events (in this case drinks consumed) in a given time interval for subject *j *in group *i*, where *f*(*Y*_*ij *_= *k*|*λ*_*ij*_) is the probability of observing *k *events. The Poisson distribution [8,10] is denoted:

P(Yij=k|λij)=exp⁡(−λij)λijkk!
 MathType@MTEF@5@5@+=feaafiart1ev1aaatCvAUfKttLearuWrP9MDH5MBPbIqV92AaeXatLxBI9gBaebbnrfifHhDYfgasaacH8akY=wiFfYdH8Gipec8Eeeu0xXdbba9frFj0=OqFfea0dXdd9vqai=hGuQ8kuc9pgc9s8qqaq=dirpe0xb9q8qiLsFr0=vr0=vr0dc8meaabaqaciaacaGaaeqabaqabeGadaaakeaacqWGqbaucqGGOaakcqWGzbqwdaWgaaWcbaGaemyAaKMaemOAaOgabeaakiabg2da9iabdUgaRjabcYha8HGaciab=T7aSnaaBaaaleaacqWGPbqAcqWGQbGAaeqaaOGaeiykaKIaeyypa0ZaaSaaaeaacyGGLbqzcqGG4baEcqGGWbaCcqGGOaakcqGHsislcqWF7oaBdaWgaaWcbaGaemyAaKMaemOAaOgabeaakiabcMcaPiab=T7aSnaaDaaaleaaieGacqGFPbqAcqGFQbGAaeaacqGFGaaicqGFGaaicqGFRbWAaaaakeaacqWGRbWAcqGGHaqiaaaaaa@526E@

for *k *= 0, 1, 2, ..., *i *= 1, 2, and *j *= 1,..., *n*_*i *_where *λ*_*ij *_> 0 and we assume that *λ*_*ij *_= *λ*_*i *_for all *j *(i.e. all subjects in a given group have the same rate of drinking). The *λ *parameter uniquely specifies this distribution, and is equal to the expected value (mean) and variance (i.e. *E*[*Y*_*ij*_] = *Var*(*Y*_*ij*_) = *λ*_*ij *_for all *i *and *j*). The maximum likelihood estimate (MLE) of λ^
 MathType@MTEF@5@5@+=feaafiart1ev1aaatCvAUfKttLearuWrP9MDH5MBPbIqV92AaeXatLxBI9gBaebbnrfifHhDYfgasaacH8akY=wiFfYdH8Gipec8Eeeu0xXdbba9frFj0=OqFfea0dXdd9vqai=hGuQ8kuc9pgc9s8qqaq=dirpe0xb9q8qiLsFr0=vr0=vr0dc8meaabaqaciaacaGaaeqabaqabeGadaaakeaaiiGacuWF7oaBgaqcaaaa@2E77@_*i *_is given by Y¯
 MathType@MTEF@5@5@+=feaafiart1ev1aaatCvAUfKttLearuWrP9MDH5MBPbIqV92AaeXatLxBI9gBaebbnrfifHhDYfgasaacH8akY=wiFfYdH8Gipec8Eeeu0xXdbba9frFj0=OqFfea0dXdd9vqai=hGuQ8kuc9pgc9s8qqaq=dirpe0xb9q8qiLsFr0=vr0=vr0dc8meaabaqaciaacaGaaeqabaqabeGadaaakeaadaqdaaqaaiabdMfazbaaaaa@2DF8@_*i*_. In this setting, the test of randomized group effects for the Poisson model is a test of the null hypothesis that *λ*_1 _= *λ*_2_.

One limitation of this model is that it may be overly simplistic and may not provide an adequate fit to consumption data of the type that we consider. The constraint that the variance is equal to the mean may lead to incorrect test results.

Consider as an example the data from the ASAP study control group at 3 months. For this dataset, non-integer count values are possible. These arise when subjects consume a number of drinks not divisible by 30 (in the case of 30-day assessments). One approach in this situation would be to model the number of drinks consumed in a 30 day period, or utilize the non-integer values. Sometimes even the 30 day value is non-integer because people report a drink size that is then translated into standard drinks. The maximum likelihood estimates of the probability distributions remains the same for non-integer values, though it is necessary to move each non-integer observed value to the next integer (using a ceiling function) to be plotted. For the models that we discuss, we can plug non-integer values into the software and still get sensible results.

**Figure 1:**
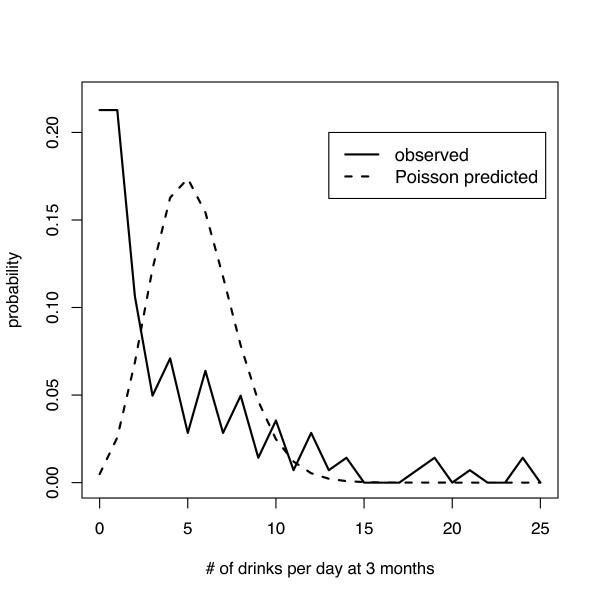
Observed value of drinks per day for the control group of the ASAP study at 3 months, plus the estimated Poisson fit to these data (λ^
 MathType@MTEF@5@5@+=feaafiart1ev1aaatCvAUfKttLearuWrP9MDH5MBPbIqV92AaeXatLxBI9gBaebbnrfifHhDYfgasaacH8akY=wiFfYdH8Gipec8Eeeu0xXdbba9frFj0=OqFfea0dXdd9vqai=hGuQ8kuc9pgc9s8qqaq=dirpe0xb9q8qiLsFr0=vr0=vr0dc8meaabaqaciaacaGaaeqabaqabeGadaaakeaaiiGacuWF7oaBgaqcaaaa@2E77@_1 _= 4.98).

Figure 1 displays the observed distribution and superimposed Poisson with λ^
 MathType@MTEF@5@5@+=feaafiart1ev1aaatCvAUfKttLearuWrP9MDH5MBPbIqV92AaeXatLxBI9gBaebbnrfifHhDYfgasaacH8akY=wiFfYdH8Gipec8Eeeu0xXdbba9frFj0=OqFfea0dXdd9vqai=hGuQ8kuc9pgc9s8qqaq=dirpe0xb9q8qiLsFr0=vr0=vr0dc8meaabaqaciaacaGaaeqabaqabeGadaaakeaaiiGacuWF7oaBgaqcaaaa@2E77@_1 _= Y¯
 MathType@MTEF@5@5@+=feaafiart1ev1aaatCvAUfKttLearuWrP9MDH5MBPbIqV92AaeXatLxBI9gBaebbnrfifHhDYfgasaacH8akY=wiFfYdH8Gipec8Eeeu0xXdbba9frFj0=OqFfea0dXdd9vqai=hGuQ8kuc9pgc9s8qqaq=dirpe0xb9q8qiLsFr0=vr0=vr0dc8meaabaqaciaacaGaaeqabaqabeGadaaakeaadaqdaaqaaiabdMfazbaaaaa@2DF8@_1 _= 4.98 using the prcounts routine in Stata [8]. The axis for the number of drinks per day after 3 months was limited to 25 drinks to improve readability (the maximum observed count was 48.6). There is a pronounced lack of fit to this model, particularly for values of less than 10 drinks per day. For the ASAP data, the assumption that the mean is equal to the variance is not tenable. In fact, the observed variance (71.7) is more than an order of magnitude larger than the mean. Also, note that there is some evidence for digit preference (even numbers are more common than odd numbers).

One approach to loosen the restrictive variance assumption involves use of an empirical (or *robust *or *sandwich*) variance estimator [11-13] to account for the over-dispersion. This more flexible extension of the Poisson allows the variance to be unconstrained. The over-dispersed Poisson option is available in a number of general purpose statistics packages (e.g. the robust option in Stata).

Another approach is to fit a negative binomial (two parameter) count model (NB) [5-8,10]. One common parametrization of the negative binomial distribution is given by:

P(Yij=k|λi,θi)=Γ(θi−1+k)Γ(θi−1)Γ(k+1)(θi−1θi−1+λi)θi−1(λiθi−1+λi)k
 MathType@MTEF@5@5@+=feaafiart1ev1aaatCvAUfKttLearuWrP9MDH5MBPbIqV92AaeXatLxBI9gBaebbnrfifHhDYfgasaacH8akY=wiFfYdH8Gipec8Eeeu0xXdbba9frFj0=OqFfea0dXdd9vqai=hGuQ8kuc9pgc9s8qqaq=dirpe0xb9q8qiLsFr0=vr0=vr0dc8meaabaqaciaacaGaaeqabaqabeGadaaakeaafaqadeGabaaabaGaemiuaaLaeiikaGIaemywaK1aaSbaaSqaaiabdMgaPjabdQgaQbqabaGccqGH9aqpcqWGRbWAcqGG8baFiiGacqWF7oaBdaWgaaWcbaGaemyAaKgabeaakiabcYcaSiab=H7aXnaaBaaaleaacqWGPbqAaeqaaOGaeiykaKIaeyypa0dabaWaaSaaaeaacqqHtoWrcqGGOaakcqWF4oqCdaqhaaWcbaGaemyAaKgabaGaeyOeI0IaeGymaedaaOGaey4kaSIaem4AaSMaeiykaKcabaGaeu4KdCKaeiikaGIae8hUde3aa0baaSqaaiabdMgaPbqaaiabgkHiTiabigdaXaaakiabcMcaPiabfo5ahjabcIcaOiabdUgaRjabgUcaRiabigdaXiabcMcaPaaadaqadaqaamaalaaabaGae8hUde3aa0baaSqaaiabdMgaPbqaaiabgkHiTiabigdaXaaaaOqaaiab=H7aXnaaDaaaleaacqWGPbqAaeaacqGHsislcqaIXaqmaaGccqGHRaWkcqWF7oaBdaWgaaWcbaGaemyAaKgabeaaaaaakiaawIcacaGLPaaadaahaaWcbeqaaiab=H7aXnaaDaaameaacqWGPbqAaeaacqGHsislcqaIXaqmaaaaaOWaaeWaaeaadaWcaaqaaiab=T7aSnaaBaaaleaacqWGPbqAaeqaaaGcbaGae8hUde3aa0baaSqaaiabdMgaPbqaaiabgkHiTiabigdaXaaakiabgUcaRiab=T7aSnaaBaaaleaacqWGPbqAaeqaaaaaaOGaayjkaiaawMcaamaaCaaaleqabaGaem4AaSgaaaaaaaa@7E18@

where Γ(·) denotes the Gamma function, *λ*_*i *_> 0 and *θ*_*i *_> 0. We note that *E*[*Y*_*ij*_] = *λ*_*i *_and *Var*(*Y*_*ij*_) = *λ*_*i *_+ λi2
 MathType@MTEF@5@5@+=feaafiart1ev1aaatCvAUfKttLearuWrP9MDH5MBPbIqV92AaeXatLxBI9gBaebbnrfifHhDYfgasaacH8akY=wiFfYdH8Gipec8Eeeu0xXdbba9frFj0=OqFfea0dXdd9vqai=hGuQ8kuc9pgc9s8qqaq=dirpe0xb9q8qiLsFr0=vr0=vr0dc8meaabaqaciaacaGaaeqabaqabeGadaaakeaaiiGacqWF7oaBdaqhaaWcbaGaemyAaKgabaGaeGOmaidaaaaa@30E1@ * *θ*_*i *_= *λ *_i _* (1 + *λ*_i _* *θ*_*i*_) for all *i *and *j *and that *Var*(*Y*_*ij*_) > *E*[*Y*_*ij*_]. It can be shown that the negative binomial can be derived in terms of a Poisson random variable where the parameter *λ*_*i *_varies according to a gamma distribution.

The negative binomial model is attractive because it allows the relaxation of strong assumptions regarding the relationship between the mean and the variance. This flexibility comes at some cost, since a two-parameter model is inherently more complicated to interpret.

Other models have been proposed that allow for an extra abundance of subjects with no consumption. In alcohol consumption outcomes, there may be subjects who are "non-susceptible" (e.g. abstinent). These "zero-inflation" (or "hurdle") models account for subjects who are structural zeros (e.g., abstinent subjects thought of as "non-susceptible") [2,3]. Conditional on being susceptible (with some probability), the distribution is assumed to be Poisson or negative binomial.

Zero-inflated Poisson (ZIP) models [3] separately estimate a parameter *p*_*i *_that governs the proportion of non-susceptible subjects in the *i*th group:

f(Yij=k|λi,pi)=   I(k=0)pi+(1−pi)exp⁡(−λi)λikk!,
 MathType@MTEF@5@5@+=feaafiart1ev1aaatCvAUfKttLearuWrP9MDH5MBPbIqV92AaeXatLxBI9gBaebbnrfifHhDYfgasaacH8akY=wiFfYdH8Gipec8Eeeu0xXdbba9frFj0=OqFfea0dXdd9vqai=hGuQ8kuc9pgc9s8qqaq=dirpe0xb9q8qiLsFr0=vr0=vr0dc8meaabaqaciaacaGaaeqabaqabeGadaaakeaafaqadeGabaaabaGaemOzayMaeiikaGIaemywaK1aaSbaaSqaaiabdMgaPjabdQgaQbqabaGccqGH9aqpcqWGRbWAcqGG8baFiiGacqWF7oaBdaWgaaWcbaGaemyAaKgabeaakiabcYcaSiabdchaWnaaBaaaleaacqWGPbqAaeqaaOGaeiykaKIaeyypa0dabaGaemysaKKaeiikaGIaem4AaSMaeyypa0JaeGimaaJaeiykaKIaemiCaa3aaSbaaSqaaiabdMgaPbqabaGccqGHRaWkcqGGOaakcqaIXaqmcqGHsislcqWGWbaCdaWgaaWcbaGaemyAaKgabeaakiabcMcaPmaalaaabaGagiyzauMaeiiEaGNaeiiCaaNaeiikaGIaeyOeI0Iae83UdW2aaSbaaSqaaiabdMgaPbqabaGccqGGPaqkcqWF7oaBdaqhaaWcbaGaemyAaKgabaGaeeiiaaIaem4AaSgaaaGcbaGaem4AaSMaeiyiaecaaiabcYcaSaaaaaa@6314@

for 0 <*p*_*i *_< 1 and *λ*_*i *_> 0 where *I*(*k *= 0) is equal to 1 when *k *= 0, and equal to 0 otherwise. By distinguishing *Always-0 *(with probability *p*_*i*_) and *Not Always-0 *group (with probability (1 - *p*_*i *_) * *exp*(-*λ*_*i*_)) for abstainers and drinkers who didn't drink during the reporting period, respectively, it can incorporate an overabundance of zeros [8]. Conditional on being a *Not Always-0*, counts are given by the Poisson distribution. This approach has been generalized to a regression framework, and implemented in general purpose statistical software (e.g. zip in Stata).

In many settings, the assumption that after accounting for the zeros the remaining counts are Poisson may not be tenable. The zero-inflated negative binomial (ZINB) allows for over-dispersion in this manner, though at the cost of more parameters.

Another approach to the modeling of count data involves use of a linear model (assuming that the observations are approximately Gaussian). While this is an extremely flexible model that is typically robust to misspecification (since the mean and variance are not linked), the linear model is less attractive because it may predict negative values of drinking given the skewness of the distribution. Use of a linear model is also inefficient if the variance is a function of the mean.

### Simulation study

To better understand the behavior of these methods in a known situation, we conducted a series of simulation studies with parameters derived from the motivating example. These simulation studies were designed to address the question of whether or not the models were robust to misspecification of the underlying count distribution. More formally, we wanted to assess whether these models preserved the appropriate Type-I error rate (the probability of rejecting the null hypothesis when it is true) when there are no true differences between groups (i.e. do they reject the null at the appropriate *α *level).

**Figure 2:**
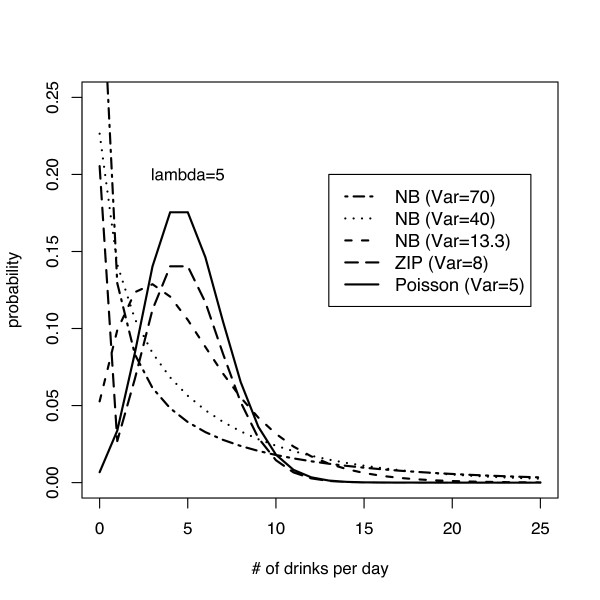
Graphical display of the five distributions, all with rate parameter 5, used in the simulations (Poisson [Var = 5], negative binomial [NB13, Var = 13], negative binomial [NB40, Var = 40], negative binomial [NB 70, Var = 70] and zero-inflated Poisson [ZIP, p = 0.2, Var = 8]).

For each set of parameters within a simulation, 100 observations were generated in each of two groups, to mimic a randomized clinical trial setting. The amount of alcohol consumption, in drinks per day was the outcome. For each simulated dataset a series of models (Poisson, negative binomial and zero-inflated Poisson) were fit. This process was repeated 2500 times for each set of parameters, where *E*[*Y*_*i*_] = *λ *= 5 (taken from the ASAP control group) and an *α *level of 0.05 was used. For the simulation of Poisson data the variance was equal to the mean. Negative binomial distributions were simulated using three arbitrary variances (13.3, 40 and 70), with the latter value comparable to the observed variance from the ASAP control group. The zero-inflated model had a probability of 0.2 of being a structural zero, and Poisson with *λ *= 5 otherwise. The true distributions for the simulations are displayed in Figure 2. Models were fit using the Poisson distribution, over-dispersed Poisson using an empirical variance estimator, negative binomial and zero inflated Poisson. We estimated the probability that each model rejected the null hypothesis and constructed a 99% confidence interval around this estimate. The code for the simulations is available upon request from the first author.

### ASAP study

The ASAP study was a randomized clinical trial of the effectiveness of a brief motivational intervention [14] on alcohol consumption among a group of hospitalized patients at Boston Medical Center. Details of the recruitment procedures, inclusion criteria, description of sample and results of the RCT have been published [15]. The Institutional Review Board of Boston University Medical Center approved this study, and the Institutional Review Board of Smith College approved the secondary analyses. After consenting to enroll, all subjects received an interviewer-administered baseline assessment prior to randomization into the control or intervention group. Subjects were randomly assigned to control or intervention group using a blocked randomization procedure. Intervention subjects participated in a brief motivational interview with a counselor (less than half an hour). Control subjects received usual care.

Follow-up was planned at 3-month and 12-month timepoints. Because the subjects came from a transient and hard-to-reach population, the researchers employed exhaustive techniques to track subjects over the follow-up period. The two primary alcohol-related outcomes were measures of alcohol consumption and linkage to appropriate alcohol treatment; for these secondary analyses we focus solely on treatment differences in alcohol consumption. The outcome of interest was the average number of standard drinks consumed per day in the past thirty days as reported using the Timeline Followback method [1] at the 3 and 12-month interviews. For the purpose of this secondary analysis we consider the 3 month time point; similar results were seen utilizing 12 month data (not reported here).

Eight models were fit comparing treatment to control for the ASAP study:

**Poisson **standard Poisson model,

**Over-dispersed Poisson **Poisson model with empirical ("robust") variance estimator,

**NB **negative binomial,

**ZIP **zero-inflated Poisson, shared inflation parameter estimated for both randomized groups (*p*_1 _= *p*_2_),

**ZINB **zero-inflated negative binomial, shared inflation parameter estimated for both randomized groups (*p*_1 _= *p*_2_),

**TTEST **two-sample unequal variance t-test,

**WILCOXON **Wilcoxon-Mann-Whitney, a non-parametric two-sample comparison procedure suitable for ordinal data, and

**PERMUTE **two-sample permutation test.

## Results

### Simulation studies

In the simulation studies we assessed the behavior of models when the null hypothesis was true (there were no differences between alcohol consumption for groups 1 and 2). We note that the ZIP model failed to converge for more than a quarter of the simulations from the standard Poisson distribution. This is likely due to the fact that many datasets had no zeros whatsoever (for the Poisson distribution with *λ *= 5, the probability that a dataset has no zeros whatsoever is equal to (1 - exp(-5))^100 ^= 0.51).

Table 1 displays the estimated Type I error rate (when there is no difference between the groups) when *α *was set to 0.05. The negative binomial model was conservative when the underlying data were zero-inflated. When the underlying distributions were not Poisson, the Poisson model did not maintain the appropriate Type I error rate. When the count models were over-dispersed by a factor of more than 2 (i.e. *Var*(*Y*_*i*_) > 2 * *E*[*Y*_*i*_]), the Poisson model rejected more than 22% of the time. When the over-dispersion was more extreme (factor of 8 and 14), the Type I error rate was 47% and 58%, respectively. The severe lack of robustness of the Poisson model in this setting is a serious concern.

**Table 1:** Estimated probability (and 99% CI) of rejecting the null hypothesis when there is no true difference between groups for a variety of statistical models and underlying distributions (results that do not include the alpha level of 0.05 are bolded)

	Analysis model fit			
True Distribution:	Poisson	ODP	NB	ZIP

Poisson (Var = 5)	.053 (.041,.064)	.054 (.042,.066)	.047 (.036,.058)	.055* (.043,.067)
NB (Var = 13)	**.225 **(.204,.247)	.049 (.038,.060)	.049 (.038,.060)	.050 (.039,.061)
NB (Var = 40)	**.467 **(.441,.493)	.047 (.036,.058)	.044 (.033,.055)	.046 (.036,.057)
NB (Var = 70)	**.584 **(.558,.609)	.052 (.041,.063)	.048 (.037,.059)	.062 (.049,.074)
ZIP (Var = 8)	**.179 **(.159,.199)	.058 (.046,.070)	**.031 **(.022,.040)	.051 (.040,.063)

all distributions except ZIP have *E*[*Y*_*i*_] = *λ *= 5, for ZIP *E*[*Y*_*i*_] = 0.8 * 5 = 4.

ODP (over-dispersed Poisson); NB (negative binomial); ZIP (zero-inflated Poisson)

* For the true distribution under the Poisson, the ZIP model failed to converge for n = 672 of the simulations.

### ASAP study

Of 341 subjects enrolled in the clinical trial, 169 subjects were randomized to the control group and the other 172 into the intervention group. The mean age of the subjects was 44.3 (SD = 10.7). Twenty-nine percent were women, 45% were Black, 39% White, 9% Hispanic, and 7% Other. Sixty-three percent were unemployed during the past three months and 25% of the subjects were homeless at one point during the past three months. Four percent of the subjects met criteria for current (past year) alcohol abuse and 77% were alcohol dependent.

We analyze the 3-month follow-up data for which 271 subjects were observed (141 control, 130 treatment), for an overall response rate of 79%. Table 2 displays the distribution of drinks per day at baseline and 3-month follow-up separately for each group. As noted earlier, drinking outcomes are highly skewed to the right, with some extremely large values. These extreme values are plausible given the large number of dependent drinkers in the sample, many of whom have developed tolerance (the need to consume large amounts of alcohol to induce effects). We also note that reported drinking quantities decreased for both groups between baseline and 3-month outcome.

**Table 2:** Distribution of drinking outcome by timepoint and randomization group

	Base line		3 Months	
	C (n = 169)	T (n = 72)	C (n = 141)	T (n = 130)

MIN	0.17	0	0	0
25th percentile	1.14	1.32	0.17	0.13
MEDIAN	3.47	3.85	1.8	1.6
75th percentile	8.23	9.12	6.1	5.7
MAX	61.77	60	48.6	38.43
mean (SD)	6.95 (9.58)	6.68 (8.44)	4.98 (8.47)	4.36 (6.47)

Table 3 displays the results from the ASAP study using a variety of count models. Use of the Poisson model yielded a statistically significant p-value, in contrast to the other methods (all other p-values > 0.45).

**Table 3:** p-values for the ASAP randomization group effect at 3 months for a variety of count models

MODEL	p-value
Poisson	.018
over-dispersed Poisson	.489
Negative binomial	.458
zero-inflated Poisson	.542
zero-inflated negative binomial	.489
t-test	.495
Wilcoxon	.805
Permutation	.746

**Figure 3:**
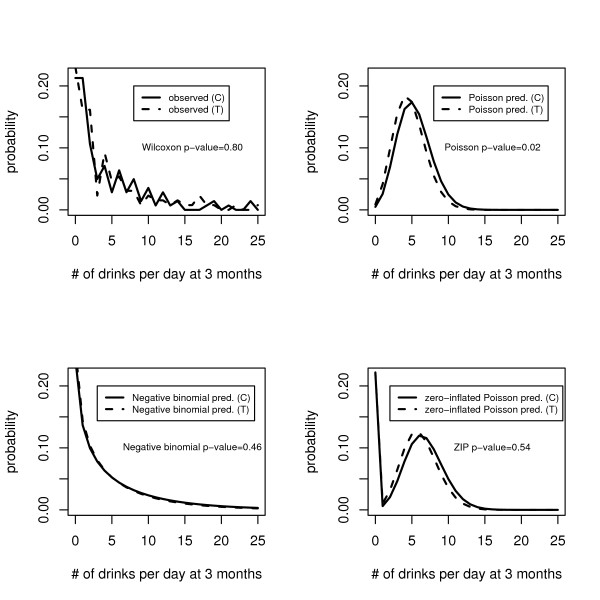
Observed and predicted values from the ASAP study at 3 months for control and treatment groups for each of four models: Wilcoxon, Poisson, negative binomial and zero-inflated Poisson.

**Figure 4:**
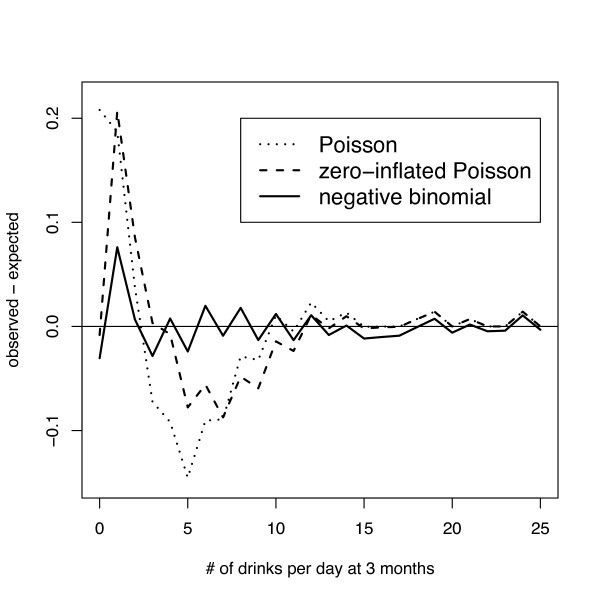
Observed minus expected values from the ASAP study at 3 months as a function of count for the Poisson, negative binomial and zero-inflated Poisson.

Figure 3 displays the observed and predicted counts for the Poisson, negative binomial, and ZIP models, while Figure 4 displays the plot of (observed minus expected) for the Poisson, negative binomial and ZIP models for the control group. The standard Poisson model provides an unsatisfactory fit, and is not appropriate for the analysis of this dataset. The fit of the zero-inflated Poisson is improved, particularly for modeling the probability of no drinking, but remains unsatisfactory over most of the remaining values. The negative binomial provides an excellent fit for these data, and that there is no indication that any further zero-inflation is needed, since the model already overpredicts zeros (hence the predicted values for the NB and ZINB would be identical).

In this setting, there was little indication from the observed plots that there were significant group differences. As seen in the simulation studies, the Poisson may not have preserved the appropriate Type I error rate due to the extremely large values of drinking for some subjects. The Appendix includes the Stata commands to fit these models and the output, along with the code to generate observed and predicted plots using the prcounts routine.

## Discussion and conclusion

A number of models have been proposed for the analysis of count data, and these models are now available in general purpose statistical packages. We have described these methods in the context of modeling reports of alcohol consumption, where a large proportion of respondents report no drinking, and a small number of respondents typically account for an extreme amount of drinking.

For the analysis of the ASAP study, we found that the standard Poisson had an extremely poor fit, and yielded a statistically significant p-value (in contrast to all of the other models, which had highly non-significant results). The unrealistic assumption that the expected rate of drinking is the same for all subjects may partially account for the poor fit of the Poisson distribution. We caution against use of the Poisson for this analysis. The negative binomial fit particularly well, and we saw no evidence for zero-inflation.

In settings where there are excess zeros, zero-inflation models are attractive. One advantage of these models is that they can estimate the probability of being a zero as a function of covariates, as well as allowing the rate parameter to be a function of covariates. In an alcohol study, the intervention may be hypothesized to affect the abstinence proportion as well as the rate parameter for drinkers. Ad-hoc methods in this setting might involve estimating the proportion of drinkers at follow-up, and in a separate model, estimating the amount of drinking amongst the subset of subjects who reported any drinking. A more principled approach involves the simultaneous estimation of the zero-inflation factor (testing *p*_1 _= *p*_2_) and the rate parameter (testing *λ*_1 _= *λ*_2_). Slymen and colleagues [2] adopted this approach by simultaneously fitting separate models for what they describe as the "logistic" component and the "Poisson" component, and this approach is also detailed in books by Winkelmann [7] as well as Cameron and Trivedi [4].

The results of the simulation studies and the secondary analyses of the ASAP study demonstrated the importance of appropriately modeling count outcomes. We caution against the use of the standard Poisson model when the mean and variance are not equal. Extensions of the Poisson (incorporating an over-dispersion parameter or use of the negative binomial distribution and/or zero-inflated models) are now available in general purpose statistical software, and address many of the shortcomings of the overly simplistic Poisson model.

As always, analysts are obliged to look at their data and utilize models that provide an appropriate fit in their situation. In particular, for models of alcohol consumption, attention should be paid to the functional form of the outcome to ensure that underlying assumptions of the methods utilized are met.

## Authors' contributions

NH conceived of the project and provided overall guidance, in addition to reviewing and interpreting analyses, and drafting the manuscript. EK participated in the drafting of the manuscript, and carried out analyses and simulations. RS led the ASAP study and participated in the drafting of the manuscript. All authors read and approved the final version of the manuscript.

## Pre-publication history

The pre-publication history for this paper can be accessed here:



## Supplementary Material

Additional File 1 Appendix. Stata code and results for count models. Click here for file
