# Supplementary material for: A cautionary note regarding count models of alcohol consumption in randomized controlled trials
Source: BMC Med Res Methodol. 2007 Feb 15;7:9. doi: 10.1186/1471-2288-7-9 (PMC1810542; doi:10.1186/1471-2288-7-9)
Supplement: Additional File 1 — Appendix. Stata code and results for count models. [file 1471-2288-7-9-S1.pdf]

A cautionary note regarding count models of alcohol consumption in randomized controlled trials (Horton et al, *BMC Medical Research Methodology*)

Appendix: Stata commands and output

Installing S-Post

SPost must be installed prior to using the `prcounts` routine:

```
. net from http://www.indiana.edu/~jslsoc/stata
. net install spost9_ado
. net get spost9_do
```

For more information, see `http://www.indiana.edu/~jslsoc/spost.htm`.

ASAP Analysis

```
. poisson drkaday treatment if tp==3

Poisson regression                                Number of obs   =        271
                                                  LR chi2(1)      =       5.62
                                                  Prob > chi2     =     0.0177
Log likelihood =  -1427.546                       Pseudo R2       =     0.0020
------------------------------------------------------------------------------
     drkaday |      Coef.   Std. Err.      z    P>|z|     [95% Conf. Interval]
-------------+----------------------------------------------------------------
   treatment |  -.1336519   .0564694    -2.37   0.018    -.2443298    -.022974
       _cons |     1.6057   .0377326    42.55   0.000     1.531746    1.679655
------------------------------------------------------------------------------


. poisson drkaday treatment if tp==3, robust

Poisson regression                                Number of obs   =        271
                                                  Wald chi2(1)    =       0.48
                                                  Prob > chi2     =     0.4890
Log pseudolikelihood =  -1427.546                 Pseudo R2       =     0.0020
------------------------------------------------------------------------------
```

```
             |               Robust
    drkaday |      Coef.   Std. Err.      z    P>|z|     [95% Conf. Interval]
-------------+----------------------------------------------------------------
   treatment |  -.1336519   .1931739    -0.69   0.489    -.5122657    .2449619
       _cons |     1.6057   .1429017    11.24   0.000     1.325618    1.885783
------------------------------------------------------------------------------


. nbreg drkaday treatment if tp==3
note: you are responsible for interpretation of non-count dep. variable

Negative binomial regression                      Number of obs   =        271
                                                  LR chi2(1)      =       0.55
Dispersion     = mean                             Prob > chi2     =     0.4588
Log likelihood = -692.23918                       Pseudo R2       =     0.0004
------------------------------------------------------------------------------
     drkaday |      Coef.   Std. Err.      z    P>|z|     [95% Conf. Interval]
-------------+----------------------------------------------------------------
   treatment |  -.1336519   .1801373    -0.74   0.458    -.4867144    .2194106
       _cons |     1.6057   .1243391    12.91   0.000        1.362    1.849401
-------------+----------------------------------------------------------------
    /lnalpha |   .6826635   .1007122                      .4852712    .8800558
-------------+----------------------------------------------------------------
       alpha |   1.979142   .1993238                      1.624616    2.411034
------------------------------------------------------------------------------
Likelihood-ratio test of alpha=0:  chibar2(01) = 1470.61 Prob>=chibar2 = 0.000


. zip drkaday treatment if tp==3, infl(_cons) robust

Zero-inflated Poisson regression                  Number of obs   =        271
                                                  Nonzero obs     =        211
                                                  Zero obs        =         60

Inflation model      = logit                      Wald chi2(1)    =       0.37
Log pseudolikelihood = -1253.585                  Prob > chi2     =     0.5424
------------------------------------------------------------------------------
             |               Robust
             |      Coef.   Std. Err.      z    P>|z|     [95% Conf. Interval]
-------------+----------------------------------------------------------------
drkaday      |
   treatment |  -.1125156   .1846819    -0.61   0.542    -.4744855    .2494544
       _cons |   1.843205   .1374971    13.41   0.000     1.573716    2.112695
-------------+----------------------------------------------------------------
inflate      |
```

```
       _cons |   -1.26964   .1481197    -8.57   0.000    -1.559949   -.9793308
------------------------------------------------------------------------------



. ttest drkaday if tp==3, by(treatment) unequal

Two-sample t test with unequal variances
------------------------------------------------------------------------------
Group |   Obs    Mean    Std. Err.  Std. Dev. [95% Conf. Interval]
--------+---------------------------------------------------------------------
    0 |   141  4.981347  .7130616  8.467138   3.571586    6.391109
    1 |   130  4.358154  .5676215  6.471881   3.235101    5.481207
--------+---------------------------------------------------------------------
combined| 271  4.682398  .4597568  7.568552   3.777234    5.587563
--------+---------------------------------------------------------------------
 diff |         .6231937 .9114006           -1.171465    2.417852
-----------------------------------------------------------------------------
diff = mean(0) - mean(1)                              t =   0.6838
Ho: diff = 0         Satterthwaite's degrees of freedom =  260.237

   Ha: diff < 0           Ha: diff != 0            Ha: diff > 0
Pr(T < t) = 0.7526   Pr(|T| > |t|) = 0.4947      Pr(T > t) = 0.2474


. ranksum drkaday if tp==3, by(treatment)

Two-sample Wilcoxon rank-sum (Mann-Whitney) test
   treatment |      obs    rank sum    expected
-------------+---------------------------------
           0 |      141     19334.5       19176
           1 |      130     17521.5       17680
-------------+---------------------------------
    combined |      271       36856       36856

unadjusted variance   415480.00
adjustment for ties    -4526.13
                     ----------
adjusted variance     410953.87

Ho: drkaday(treatm~t==0) = drkaday(treatm~t==1)
            z =   0.247
    Prob > |z| =   0.8047
```

```
. permute random2 r(sum), reps(10000) nodots nodrop nowarn: summarize drkaday if treatment==1

Monte Carlo permutation results        Number of obs   =        271

      command:  summarize drkaday if treatment==1
        _pm_1:  r(sum)
  permute var:  random2
------------------------------------------------------------------------------
T        |   T(obs)     c      n    p=c/n   SE(p)  [95% Conf. Interval]
---------+--------------------------------------------------------------------
   _pm_1 |   566.56   7461  10000  0.7461  0.0044  .7374485   .7546079
------------------------------------------------------------------------------
Note:  Confidence interval is with respect to p=c/n.
Note:  c = #{|T| >= |T(obs)|}
```

Plotting predicted values

```
. use "asap.dta", clear
. gen drkinteger = ceil(drkaday)
(124 missing values generated)
. poisson drkinteger treatment if tp==3

Poisson regression                          Number of obs   =        271
                                            LR chi2(1)      =       4.75
                                            Prob > chi2     =     0.0293
Log likelihood = -1398.9603                 Pseudo R2       =     0.0017
-----------------------------------------------------------------------------
 drkinteger|      Coef.   Std. Err.    z   P>|z|    [95% Conf. Interval]
----------+------------------------------------------------------------------
 treatment |  -.1182639    .054343 -2.18  0.030  -.2247743   -.0117536
     _cons |   1.673976   .0364662 45.90  0.000   1.602504    1.745449
-----------------------------------------------------------------------------

. prcounts drkp if treatment==0, max(25) plot
(567 missing values generated)
. label var drkpobeq "Observed"
. label var drkppreq "Poisson Predicted"
. label var drkpval "# of drinks per day after 3 months"
. graph twoway connected drkpobeq drkppreq drkpval,
  msymbol(o s) title(Figure 1.) ytitle("Probability")
  ylabel(0(.1).3) xlabel(0(1)25)
```
